# Time Relationship between the Occurrence of a Thromboembolic Event and the Diagnosis of Hematological Malignancies

**DOI:** 10.3390/cancers16183196

**Published:** 2024-09-19

**Authors:** Jarosław Kępski, Sebastian Szmit, Ewa Lech-Marańda

**Affiliations:** 1 Department of Cardio-Oncology, Centre of Postgraduate Medical Education, 01-813 Warsaw, Poland; jaroslaw.kepski@gmail.com; 2 Department of Hematology, Institute of Hematology and Transfusion Medicine, 02-776 Warsaw, Poland; emaranda@ihit.waw.pl

**Keywords:** cardio-oncology, hemato-oncology, hematological malignancies, thromboembolism, myocardial infarction, stroke, pulmonary embolism

## Abstract

**Simple Summary:**

The development of cancer is associated with coagulation disorders. Blood cancers should be particularly associated with hypercoagulability and thus thromboembolic complications. An essential finding of this study is that arterial thromboembolism (myocardial infarction or stroke) occurs significantly more often than venous thromboembolism (pulmonary embolism or/and thrombosis) preceding the diagnosis of hematological malignancy. Interestingly, venous thromboembolism occurs significantly more often in acute leukemia (myeloid or lymphocytic) and chronic myeloproliferative disease. However, the chance of venous thromboembolism is increased before and after the diagnosis of myeloproliferative disease. More research is needed on the relationship between venous thromboembolism development and myeloproliferative disease progression.

**Abstract:**

Objectives. Venous and arterial thromboembolism (VTE/ATE) often coexist with onco-hematologic diagnosis. This study aimed to assess the time relationship between the diagnosis of VTE/ATE and blood cancers. The second aim was to identify VTE/ATE risk factors related to the type of hematology disease and cardiac history. Methods. A total of 1283 patients underwent cardio-oncology evaluation at the Institute of Hematology and Transfusion Medicine in Warsaw from March 2021 through March 2023 (2 years), and 101 (7.8%) cases were identified with VTE/ATE. Results. ATE compared with VTE significantly occurred more often before the diagnosis and treatment of hematologic malignancy: 33/47 (70.2%) vs. 15/54 (27.8%), *p* < 0.0001. The risk of a VTE episode is exceptionally high in the first months after the diagnosis of an onco-hematological disease and the initiation of anticancer treatment. The higher frequency of VTE was associated with acute myeloid leukemia (17 cases/270 patients/6.30%/*p* = 0.055), acute lymphocytic leukemia (7 cases/76 patients/9.21%/*p* = 0.025), and chronic myeloproliferative disease (7 cases/48 patients/14.58%/*p* = 0.0003). Only the risk of VTE was significantly increased before (OR = 6.79; 95% CI: 1.85–24.95; *p* = 0.004) and after diagnosis of myeloproliferative disease (OR = 3.12; 95% CI: 1.06–9.16; *p* = 0.04). Conclusions. ATEs occur more often than VTE before a diagnosis of blood cancer. The risk of VTE is exceptionally high before and after diagnosis of chronic myeloproliferative disease.

## 1. Introduction

A thromboembolic event may be the first symptom of cancer [1,2]. The first data on such a relationship concerned pulmonary embolism and deep vein thrombosis [3]. Another correlation was found for stroke [4,5,6]. The effects of cancer-screening programs in patients after pulmonary embolism have been analyzed [7]. Cancer-screening programs for stroke patients have also been planned [8]. We need data on the importance of myocardium infarction as the first symptom of cancer [9].

It seems that there is a high risk of thromboembolism in the group of blood cancers, where coagulation disorders may be particularly turbulent [10]. However, initiating anticancer therapy may be associated with increased activation of prothrombotic factors or damage to the vascular endothelium, consequently leading to coagulopathy. An exceptionally high risk of venous and arterial thromboembolic events (VTE/ATE) is associated with the use of antiangiogenic drugs, second- and third-generation BCR-ABL inhibitors, and immune checkpoint inhibitors [11,12,13,14]. These observations allowed us to define a separate group of complications related to oncological treatment called vascular toxicity [15]. It includes symptomatic and asymptomatic venous and arterial thrombosis, myocardial infarction, stroke, peripheral artery diseases, and coronary artery spasms.

Both venous and arterial thromboembolic events are associated with worse prognosis in cancer patients [16,17]. It seems clinically essential to assess to what extent a given event is related to the cancer and to what extent it is related to its treatment. Identification of factors predisposing to the occurrence of venous and arterial thromboembolic events and the time relationship to the moment of cancer diagnosis seems to be crucial to optimize recommendations for primary and secondary prevention. This is especially important because these patients are at an exceptionally high risk of bleeding, which results from thrombocytopenia, platelet dysfunction, and bone marrow suppression [18,19]. Moreover, knowing such a temporal relationship may help plan optimal oncological screening. This is particularly important in patients with blood cancers because we have the most minor epidemiological data.

The aim of this study was to assess the time relationship between the moment of diagnosis of hematological cancer and the moment of occurrence of venous or arterial thromboembolism. The second aim was to identify VTE/ATE risk factors related to the type of cancer and cardiac characteristics of the patients.

## 2. Materials and Methods

The retrospective analysis included patients evaluated by cardio-oncologists certified by the International Cardio-Oncology Society at the reference Polish hematology center—the Institute of Hematology and Transfusion Medicine in Warsaw. Clinical data were collected from March 2021 through March 2023 (2 years).

During cardio-oncology evaluation, the moment of de novo diagnosis of venous or arterial thromboembolism (VTE/ATE) was analyzed relative to the date of diagnosis of blood cancer. The second step was to assess the odds ratio for VTE/ATE depending on the type of hematologic malignancy and cardiac history data.

Statistical analyses were performed by using Statistica software (v13.3). Patient characteristics were presented as frequency of diagnoses and their percentages in population for hematologic malignancies and comorbid cardiovascular diseases, including diagnoses of venous and arterial thromboembolisms. The following were considered thromboembolic (TE) diseases: pulmonary embolism and/or thrombosis of the veins of the limbs; and the following are arterial TE: myocardial infarction and/or ischemic stroke. The chi-square test was used to compare the difference in the incidence of arterial and venous thromboembolism (ATE vs. VTE) before the diagnosis of hematologic malignancy. Then, the chi-square test with the possible Yates correction was used to determine the relationship between the more frequent occurrence of venous or arterial thromboembolism in correlation with the diagnosis of a specific type of blood cancer. Finally, logistic regression analyses were used, and the odds ratio for the occurrence of ATE and VTE was calculated depending on the type of cancer and comorbid cardiovascular diseases. Statistically significant results were considered those for which p was less than 0.05.

## 3. Results

**Figure 1:**
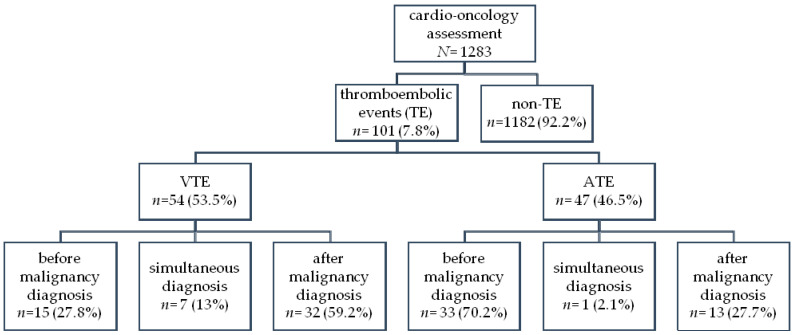
General diagram of frequency of thromboembolic events (TE): arterial thromboembolic events (ATEs) were more frequent before malignancy diagnosis than venous thromboembolic events (VTEs): 33/47 (70.2%) vs. 15/54 (27.8%), *p* < 0.0001.

At the Institute of Hematology and Transfusion Medicine in Warsaw, from March 2021 through March 2023 (two years), 1283 hemato-oncology patients underwent cardio-oncology evaluation. The majority were men (673/52.5%), and the median age was 63 years (IQ: 47–71) (Table 1, Figure 1).

The dominant hematological diagnoses were AML (*n* = 270, 21%), NHL (*n* = 250, 19.5%), MM (*n* = 223, 17.4%), CLL (*n* = 101, 7.88%), and ALL (*n* = 76, 5.92%). Nearly half of the patients had at least one comorbid cardiovascular disease, of which arterial hypertension (40%) and cardiac arrhythmias (15.8%) were predominant.

The group of 101 (7.8%) patients with a history of thromboembolism were identified in the analyzed population. VTE accounted for 53%. It was shown that VTE episodes occurred significantly more often after the initiation of anticancer treatment (*p* < 0.0001) compared with ATE, most of which were observed before (70.2%) the hematological malignancy was revealed.

**Figure 2:**
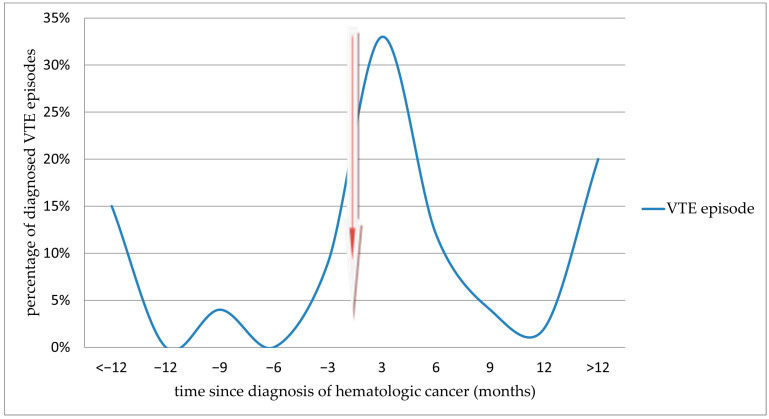
Incidence of acute VTE before and after diagnosis of hematologic malignancy. The arrow indicates the time of cancer diagnosis.

**Figure 3:**
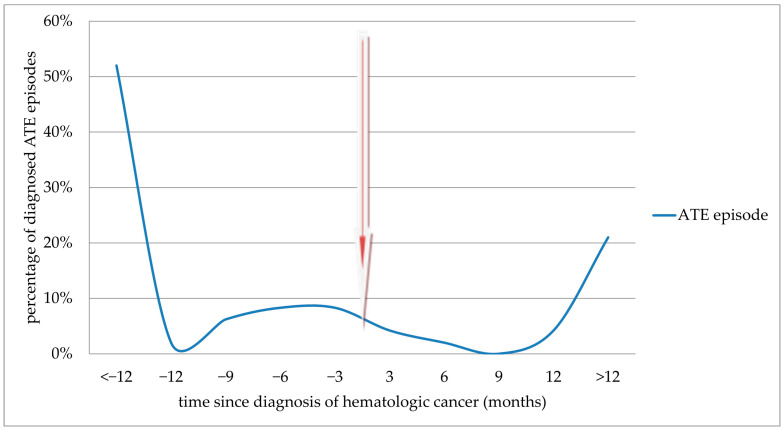
Incidence of acute ATE before and after diagnosis of hematologic malignancy. The arrow indicates the time of cancer diagnosis.

The results of the time-dependence analysis showed that the highest risk of diagnosing acute VTE occurs in the first months after starting anticancer treatment (Figure 2). A similarly strong correlation was not demonstrated in the case of arterial events, which were observed mainly before the diagnosis of hematologic cancer and thus before the initiation of anticancer treatment—33 cases, representing 70.2% of all diagnoses of arterial thromboembolic complications (Figure 3).

Through an analysis of the distribution of thromboembolic events in relation to histopathological diagnoses of hematological malignancies, it was shown that VTE was most often diagnosed in patients with AML (17 cases/6.30%/*p* = 0.055), ALL (7 cases/9.21%/*p* = 0.025), and chronic myeloproliferative disease (7 cases/14.58%/*p* = 0.0003). However, there was no increased incidence of ATE depending on the diagnosis of hematologic cancer (Table 2).

For the occurrence of ATE events before the diagnosis of hematologic cancer, the cardiac history was important both in terms of the presence of classic risk factors for atherosclerosis, i.e., older age and hypertension; and as co-occurring heart failure, coronary artery disease, or arrhythmia (Table 3). Interestingly, all these factors were significant only before the diagnosis of cancer; their significance disappeared after the diagnosis of cancer and the initiation of hematological treatment. Only coronary artery disease retained its importance for the risk of ATE after a cancer diagnosis.

Cardiological history was of no significance in the context of VTE (Table 4). Only diabetes mellitus had significant correlation with the diagnosis of VTE especially after the diagnosis of hematological malignancy (OR = 2.42, *p* = 0.03). Some histopathological diagnoses were also important for VTE. The diagnosis of acute lymphocytic leukemia correlated later with increased probability of VTE (OR = 3.05, *p* = 0.015). In the case of myeloproliferative disease, the risk of VTE was increased both before and after diagnosis (OR = 6.79, *p* = 0.004 and OR = 3.12, *p* = 0.04.

## 4. Discussion

VTE and ATE occur with quite significant frequency in hematological patients. The latest data indicate that the pathogenesis and clinical picture may differ from solid tumors [20]. Regardless of the cause or the type of tumor, the prognosis of patients with a thrombotic event is always poorer [16,21]. The mortality risk is particularly high when cancer is diagnosed at the same time as a thromboembolic event. The benefits of anticoagulant therapy using anticoagulants that are not vitamin K antagonists may also differ in hemato-oncology, and there is undoubtedly less evidence for its effectiveness [22]. There may be a greater risk of complications such as bleeding, which may be caused by thrombocytopenia and platelet dysfunction [23]. The risk of late complications such as thromboembolic pulmonary hypertension may also be higher [24]. Myeloproliferative diseases are a known risk factor for the development of pulmonary hypertension [25]. Risk factors for the development of pulmonary hypertension in the course of myeloproliferative diseases are constantly discussed [26]. Our publication shows an increased risk of venous thromboembolic complications, adding a new aspect related to thromboembolic changes. Currently, pulmonary hypertension due to myeloproliferative diseases is classified in group 5, i.e., with unknown or multifactorial pathogenesis [27].

MM has well-defined indications for primary thromboprophylaxis [28,29]. This is due to the high risk of thromboembolic events associated with the use of immunomodulatory drugs, steroids, or the presence of fractures and immobilization [30]. Lymphoma patients also usually receive primary antithrombotic prophylaxis because, according to the thromboembolic risk stratification proposed by Khorana, lymphoma receives one point [31]. Such prophylaxis can be considered from two points of view, according to the latest research [32,33]. For this reason, perhaps in our population, patients with myeloma and non-Hodgkin’s lymphoma did not experience VTE very often. 

Our data, however, confirm other reports indicating that patients with AML and ALL are at risk of VTE [14]. The results of our analysis show that patients with AML and ALL are at exceptionally high risk of VTE events. In the literature, the incidence of VTE in AML is estimated to range from 2% to nearly 15%; in our population, 6.3% of patients with AML experience an acute episode of VTE [34]. The pathogenesis of these events is complex, but considering the correlation with the initiation of chemotherapy, it should be assumed that the dominant mechanism is the massive release of procoagulants due to the lysis of cancer cells [35]. Hemodynamic changes and vascular damage strengthen this mechanism. Another factor of thrombotic events in AML is DIC (disseminated intravascular coagulation), which occurs in about 30% of patients at diagnosis [36]. The coexistence of increased risk of thrombosis and bleeding, such as thrombocytopenia typical of AML, makes it challenging to choose the optimal antithrombotic therapy. It should be emphasized that, unlike patients with lymphoma and ALL, a VTE episode in patients with AML is not associated with an increased risk of death within one year [37,38]. Our study confirmed that the problem of VTE in AML is noticeable, although the result of the correlation between VTE and AML was on the verge of statistical significance.

Another group of our patients at a clear significant risk of VTE is patients with ALL, with an incidence of 9.2%. In a large meta-analysis of 72 prospective studies, including 9061 patients, the VTE incidence rate (IR) in ALL was determined to be 5% (95% CI: 4–6%) [32]. The authors emphasize that antithrombotic prophylaxis using LMWH significantly reduces the risk of VTE events in this group. One of the main factors associated with a higher rate of thrombotic events is the use of chemotherapy [39]. There are no similar observations in the case of targeted treatment with TKIs (tyrosine kinase inhibitors), and reports regarding the use of steroids are unclear [39,40,41]. Our study clearly shows that after the diagnosis of ALL, and therefore during active treatment of ALL, we had a problem with a significantly higher incidence of VTE. Therefore, future studies should be conducted to identify risk factors for VTE in ALL related not so much to the patients’ characteristics but to the drugs used for ALL treatment and the possible degree of aggressiveness of the ALL course.

It should be added that it is surprising that patients with AML and ALL were not included in large clinical trials with new anticoagulants [42,43]. The latest guidelines of the European Society of Cardiology emphasize this [44]. This may be surprising in the context of the results of our observation and the cited studies. These patients are disqualified from large clinical trials due to thrombocytopenia and a high baseline risk of bleeding. Therefore, personalized trials should be planned for AML and ALL patients in terms of both primary and secondary prevention of VTE.

A lot of data show that the risk of both venous and arterial thromboembolic events is the highest during the period of diagnosis of cancer [45,46]. The disease in the metastatic phase poses a significantly higher risk of both venous and arterial complications, but this applies to solid tumors [14,21]. Also, recurrence of venous thromboembolism is more common in metastatic disease [47]. Additionally, the molecular type of cancer increases the risk of thromboembolism [48]. The type of anticancer therapy or supportive care is also important [49,50]. 

In hematology, of course, we cannot talk about metastatic disease, but we can use other criteria to assess the advancement or aggressive course of hematological cancer. It is worth checking to what extent VTE diagnosed, especially in ALL and myeloproliferative diseases, is associated with a negative cardiological and oncological prognosis. Our observation shows one more problem. ATE may be a clinical indicator preceding the diagnosis of hematologic cancer by up to several months. This correlation shown in Figure 3 should attract the attention of interventional cardiologists, who should monitor especially older patients after an ATE episode for a possible subsequent diagnosis of hematologic malignancy.

Our publication is the first to comprehensively analyze the problem of venous and arterial thromboembolic events in patients with hematological malignancies. We have proven that the type of histopathological diagnosis is essential for the risk of venous thromboembolic events (VTEs). Many guideline documents indicate that one should remember patient-related factors among the risk factors for thromboembolic complications and those related to cancer and cancer treatment. This, of course, includes the issue of comorbidities that may additionally increase the risk of thromboembolism. Our study shows that coexisting cardiac diseases are not crucial for the occurrence of VTE. This suggests that the main predictors of a VTE are the onco-hematological disease and/or its treatment [17,51]. Still, they are decisive for the occurrence of arterial complications, especially before cancer diagnosis. Only coronary artery disease remains a significant risk factor for ATE if hematological cancer is already diagnosed and treated. Moreover, in the aspect of hematological malignancies, only ATEs may precede the diagnosis of cancer. Some studies have shown an increased risk of ATE in the period preceding the diagnosis of cancer, suggesting that cancer is associated with a risk of ATE that goes beyond conventional cardiovascular risk factors. Further population studies are needed to determine whether patients with a history of ATE should be screened for hematological malignancies.

To some extent, our study constitutes another step in the discussion about the importance of the so-called “reverse cardio-oncology” [52,53]. This is a relatively new hypothesis claiming that cardiovascular diseases contribute to the development and progression of cancer [54]. Of course, the common denominator may be common risk factors, chronic inflammation, and tissue hypoxia in patients with atherosclerosis [55,56]. Several preclinical studies are being conducted to elucidate possible pathomechanisms [57]. Our study may complement the current discussion on the co-occurrence of heart diseases and cancer [58]. We have proven that ATEs occur quite frequently before the diagnosis of a hematologic malignancy. We do not know whether this is an early effect of clotting disorders caused by developing blood cancer.

### Limitation

Our study is not a large population study but only an observational study based on cardio-oncologists’ activities at the main hematology Polish center. However, given that the current guidelines on cardio-oncology published in 2022 by the European Society of Cardiology, in cooperation with the European Hematology Association, recommend risk stratification according to the Heart Failure Association—International Cardio-Oncology Society (HFA-ICOS) tool in patients before anticancer treatment, it can be assumed that we analyzed a representative population of patients with hematologic malignancies in the reference Polish hematology center [59]. It should be clearly highlighted that it is a cardio-oncology study without a control group without cancer diseases. Undoubtedly, large population studies are necessary to confirm our observations.

In our study, we presented the time-dependent relationship between the occurrence of a VTE or ATE episode and the diagnosis of blood cancer and the initiation of onco-hematological treatment. We also assessed the predictive value of cardiovascular diseases in the occurrence of thrombotic episodes. However, in this research, we do not analyze non-cardiac risk factors, such as obesity, low performance status, immobilization, or dehydration. However, we have proven how crucial thrombotic vigilance is in the first period after starting onco-hematological treatment. Although we did not analyze the impact of specific cancer therapy, we highlighted the prothrombotic problem especially at the beginning of treatment of acute lymphocytic leukemia and chronic myeloproliferative disease, which has not been revealed in such a significant way earlier.

## 5. Conclusions

ATEs occur significantly more often than VTEs before the diagnosis of hematological malignancies, especially in patients with a significant cardiac history.

The occurrence of VTE is most frequently observed in the first months after the diagnosis of blood cancer and the initiation of anticancer treatment. It is most closely associated with the diagnosis of acute lymphocytic leukemia and myeloproliferative disease.

## Figures and Tables

**Table 1:** Demographic characteristics of the study group of 1283 patients with hematological malignancies who underwent cardio-oncology assessment between March 2021 and March 2023.

		Number	Percentage
Sex	Men	673	52.46
Older age	≥70 years	390	30.4
Hematological malignancies	AML	270	21.04
	NHL	250	19.49
	MM	223	17,38
	CLL	101	7.87
	ALL	76	5.92
	MDS	70	5.46
	HL	56	4.36
	CML	51	3.98
	Chronic myeloproliferative disease	48	3.74
	Other rarer (together)	138	
Thromboembolism	All	101	7.87
	VTE	54	4.21
	ATE	47	3.66
Coexisting diseases	Hypertension	521	40.61
	Arrhythmia	203	15.82
	AF	105	8.18
	HF	142	11.07
	IHD	107	8.34
	DM	128	9.98

AML—acute myeloid leukemia, NHL—non-Hodgkin’s lymphoma, CLL—chronic lymphocytic leukemia, ALL—acute lymphocytic leukemia, MDS—myelodysplastic syndrome, HL—Hodgkin lymphoma, CML—chronic myeloid leukemia, VTE—venous thromboembolism, ATE—arterial thromboembolism, AF—atrial fibrillation, HF—heart failure, IHD—ischemic heart disease, DM—diabetes mellitus.

**Table 2:** Analysis of association between thromboembolism occurrence and different diagnoses of hematological malignancies.

	VTE		ATE	
	Frequency	*p*-Value	Frequency	*p*-Value
All	54 of 1283 4.21%	-	47 of 1283 3.66%	-
AML	17 of 270 6.30%	0.055	8 of 270 2.96%	0.491
NHL	7 of 250 2.80%	0.216	6 of 250 2.40%	0.236
MM	10 of 223 4.48%	0.822	11 of 223 4.93%	0.267
CLL	3 of 101 2.97%	0.698 *	6 of 101 5.94%	0.204
ALL	7 of 76 9.21%	0.025	2 of 76 2.63%	0.858 *
MDS	1 of 70 1.43%	0.376 *	3 of 70 4.29%	0.966 *
HL	0 of 56 0%	0.206 *	1 of 56 1.79%	0.688 *
CML	0 of 51 0%	0.241 *	4 of 51 7.84%	0.215 *
Chronic myeloproliferative disease	7 of 48 14.58%	0.0003	4 of 48 8.33%	0.173 *

* Yates corrected chi-square.

**Table 3:** Odds ratio for the occurrence of ATE in relation to cardiac history.

	All ATE	ATE before Diagnosis of Malignancy	ATE after Diagnosis of Malignancy
Older age (≥70 y)	OR = 3.55 (1.96–6.44) *p* = 0.00003	OR = 4.17 (2.03–8.57) *p* = 0.0001	NS
Hypertension	OR = 4.5 (2.31–8.76) *p* < 0.00001	OR=5.67 (2.44–13.16) *p* = 0.00005	NS
Arrhythmia	OR = 2.61 (1.39–4.92) *p* = 0.003	OR = 3.17 (1.53–6.55) *p*=0.002	NS
AF	NS	NS	NS
HF	OR = 3.28 (1.69–6.38) *p* = 0.0004	OR = 4.92 (2.37–10.24) *p* = 0.00002	NS
IHD	OR = 83.74 (37.63–186.32) *p* < 0.00001	OR = 108.94 (37.32–317.98) *p* < 0.00001	OR = 30.21 (9.29–98.2) *p* < 0.00001
DM	NS	NS	NS

Legend: NS—nonsignificant.

**Table 4:** Odds ratio for VTE in time relation of diagnoses of hematological malignancies.

	All VTE	VTE before Diagnosis of Malignancy	VTE after Diagnosis of Malignancy
AML	NS	NS	NS
ALL	OR = 2.5 (1.09–5.75) *p* = 0.03	NS	OR = 3.05 (1.24–7.53) *p* = 0.015
Chronic myeloproliferative disease	OR = 4.32 (1.84–10.13) *p* = 0.0008	OR = 6.79 (1.85–24.95) *p* = 0.004	OR = 3.12 (1.06–9.16) *p* = 0.04
Older age (≥70 y)	NS	NS	NS
Hypertension	NS	NS	NS
Arrhythmia	NS	NS	NS
AF	NS	NS	NS
HF	NS	NS	NS
IHD	NS	NS	NS
DM	OR = 2.43 (1.22–4.85) *p* = 0.01	NS	OR = 2.42 (1.09–5.38) *p* = 0.03

Legend: NS—nonsignificant.

## Data Availability

All data of this study will be available upon reasonable request and with permission of the Directors of the Institute of Hematology and Transfusion Medicine in Warsaw, Poland.
